# Case Report: Esophageal Bronchus in a Neonate, With Image, Histological, and Molecular Analysis

**DOI:** 10.3389/fped.2021.707822

**Published:** 2021-07-09

**Authors:** Stephen L. Trisno, Nara S. Higano, Dan Kechele, Talia Nasr, Wendy Chung, Aaron M. Zorn, Jason C. Woods, James M. Wells, Paul S. Kingma

**Affiliations:** ^1^Division of Neonatology and Pulmonary Biology, Cincinnati Children's Hospital Medical Center, Cincinnati, OH, United States; ^2^Center for Pulmonary Imaging Research, Imaging Research Center and Department of Pulmonary Medicine, Cincinnati Children's Hospital Medical Center, Cincinnati, OH, United States; ^3^Division of Developmental Biology, Center for Stem Cell and Organoid Medicine (CuSTOM), Perinatal Institute, Cincinnati Children's Hospital Medical Center, Cincinnati, OH, United States; ^4^Departments of Pediatrics and Medicine, Columbia University Irving Medical Center, New York, NY, United States; ^5^Department of Pediatrics, University of Cincinnati College of Medicine, Cincinnati, OH, United States; ^6^Division of Endocrinology, Cincinnati Children's Hospital Medical Center, Cincinnati, OH, United States

**Keywords:** esophagus, trachea, fistula, MRI, foregut malformation

## Abstract

In this case report, we describe the clinical course of a neonate who presented initially with respiratory distress and later with choking during feeding. He was subsequently found to have an esophageal bronchus to the right upper lung lobe, a rare communicating bronchopulmonary foregut malformation. Histological and molecular analysis of the fistula and distal tissues revealed that the proximal epithelium from the esophageal bronchus has characteristics of both esophageal and respiratory epithelia. Using whole exome sequencing of the patient's and parent's DNA, we identified gene variants that are predicted to impact protein function and thus could potentially contribute to the phenotype. These will be the subject of future functional analysis.

## Introduction

Of the different types of foregut malformations that can occur in embryonic development, communicating bronchopulmonary foregut malformations (CBPFM) are rare and are defined as a fistula connecting the esophagus or stomach to an isolated portion of the respiratory tract. This isolation of an entire lung, lobe, or segment is termed pulmonary sequestration (1). An esophageal bronchus is a subtype of CBPFM where a lobar bronchus connects with the esophagus, which leads to opacification and consolidation of the affected lobe (1, 2). The anomalous connections characterized in prior work have histologically briefly described the epithelium, but the true identity and developmental origins of the pathological tissue is unclear (3).

Here, we describe a neonate who presented with poor feeding and choking during feeding, was found to have normal work of breathing but diminished right upper lung sounds, and was found to have an esophageal bronchus. The pre-surgical anatomy was examined with a novel, safer 3D ultrashort echo time (UTE) MRI approach. In addition, we performed histological and molecular analysis of the fistula and isolated lung/respiratory tissue. These analyses demonstrated that the pathological tissue has characteristics of both esophageal and respiratory epithelia, and limited genetic analysis generated a list of gene variants predicted to impact protein function that may contribute to this patient's phenotype.

## Case

**Figure 1:**
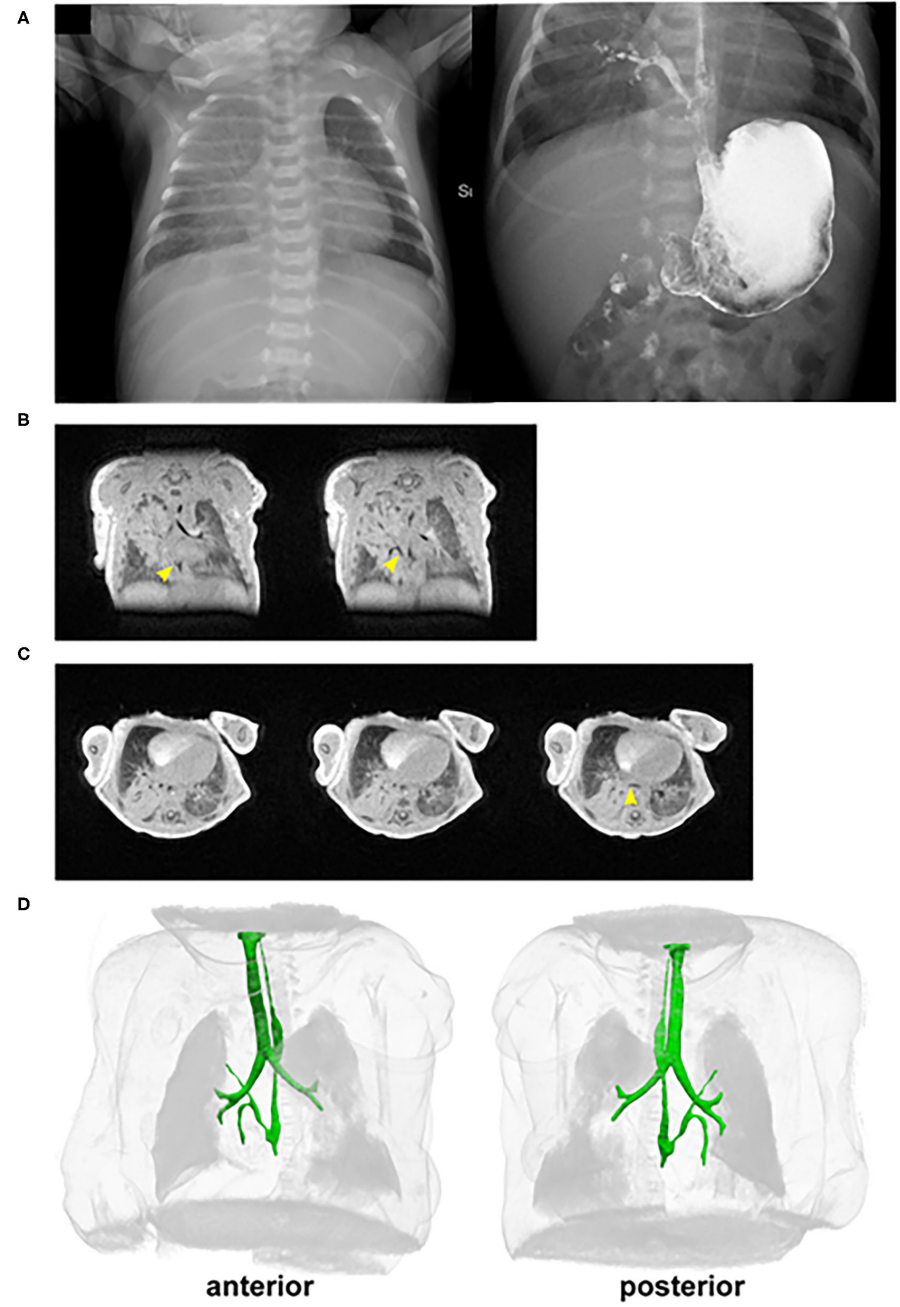
*In vivo* imaging of the patient with an esophageal bronchus. **(A)** Pre-repair/resection of (left) chest X-ray and (right) esophageal contrast study reveals opacification of the right upper lobe and the presence of an esophageal bronchus. **(B,C)** Selected **(B)** coronal and transverse **(C)** cross-sections from UTE MRI. Yellow arrowheads point to the fistula connecting from the esophagus to the right upper lobe of the lung. **(D)** 3D rendering of the chest from UTE MRI. The foregut (esophagus, trachea, and bronchi) is highlighted in green. The esophageal bronchus is seen with a superior branch proceeding to the right upper lobe. There is also a caudal branch inferior to the crossing of the right main stem pulmonary artery that is narrowed shortly after branching. The narrowing does not appear to be a result of compression by the right main stem pulmonary artery and is presumed to most likely be an intrinsic stricture.

A 3,885-g term (38-week) male was born to a 24-year-old G4P2222 female by Cesarean delivery for failure to progress. The maternal medical history was significant for diabetes of unspecified type. The prenatal course was significant for polyhydramnios; otherwise, prenatal labs and 20-week fetal ultrasound screening were normal. Apgar scores were 5 at 1 min and 6 at 5 min. The delivery was complicated by nuchal and body cord and respiratory distress requiring positive pressure ventilation, and the neonate was subsequently transferred to the neonatal intensive care unit for respiratory support with a high-flow nasal canula. After 1 day, he was weaned off oxygen and tolerating feeds, and he was discharged on day of life 3. The patient then presented to Cincinnati Children's Hospital on day of life 11 with concerns of poor feeding and choking during feeding. He was found to have right upper lobe consolidation on chest X-ray (Figure 1A) and was started empirically on ampicillin and cefotaxime with concerns for pneumonia, although urine, blood, and cerebrospinal fluid (CSF) cultures were all negative. Over the next 2 days, an esophageal contrast study (Figure 1A), 3D UTE chest MRI, and chest CT revealed a broncho-esophageal fistula arising from the distal esophagus to a segment of the right upper lobe (Figures 1B–D) (4, 5). Subsequently, a right upper lobectomy and resection of the esophageal bronchus was done on day of life 13.

After surgery, the infant remained intubated for 1 day, was weaned to high-flow nasal canula on post-operative day (POD) 1, nasal canula by POD 9, and on room air POD 15. During this time, an upper gastrointestinal contrast study demonstrated successful repair without leak but incidentally found a malrotation without obstruction and the presence of a small hiatal hernia. During the admission, he also underwent additional evaluation for vertebral defects, anal atresia, cardiac defects, tracheo-esophageal fistula, renal anomalies, and limb (VACTERL) abnormalities, including an echocardiogram and additional ultrasound imaging, which were all normal except for a horseshoe kidney. The infant tolerated feeds and was discharged, although he returned 5 months later with obstruction and underwent a Ladd's procedure to repair the malrotation.

**Figure 2:**
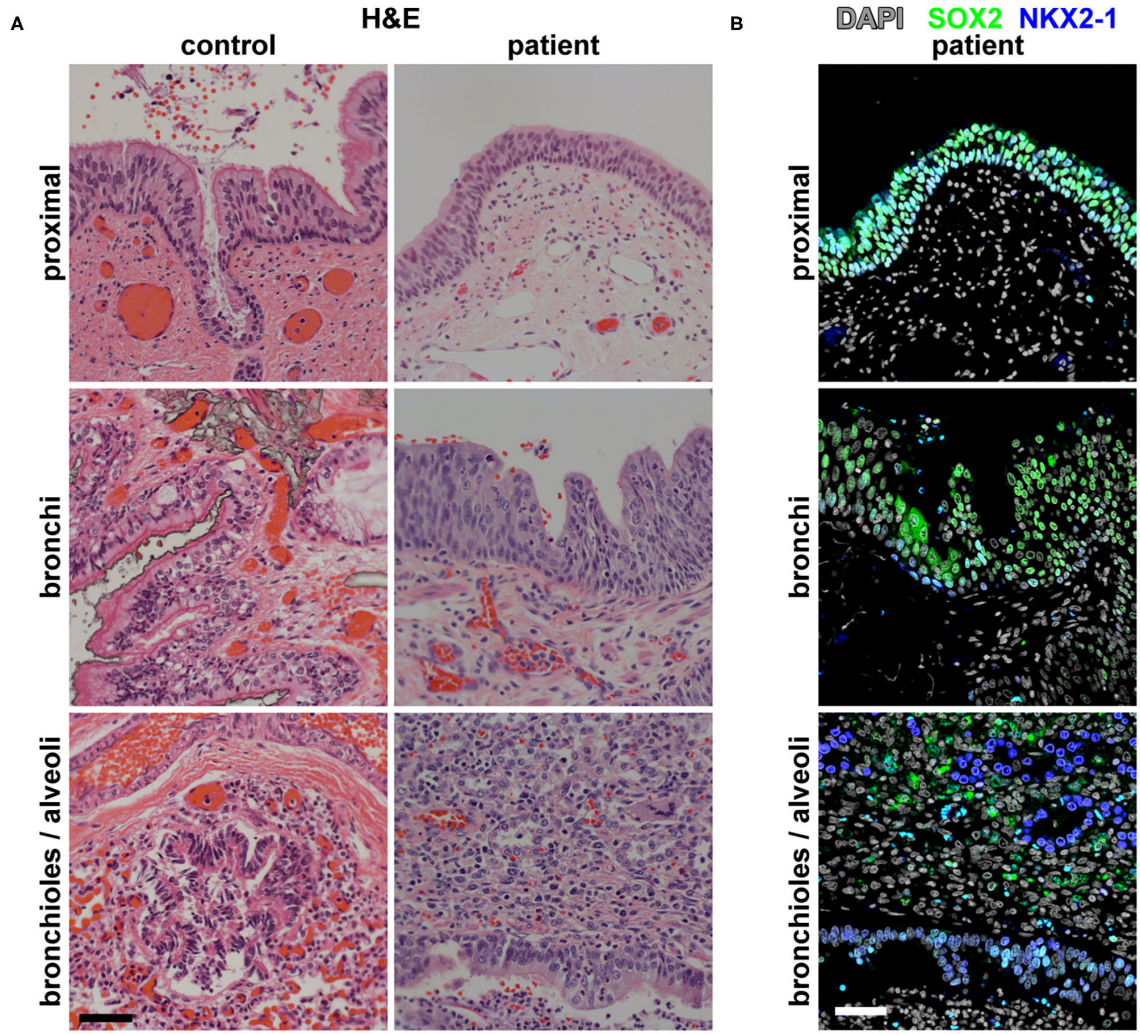
Histological and molecular analysis of the identity of the broncho-esophageal fistula and more distal airway. **(A)** H&E staining of the patient vs. control tissues from the “proximal” epithelium (broncho-esophageal fistula vs. trachea, respectively), bronchi, and distal bronchioles and alveoli. **(B)** Immunofluorescence staining of patient tissue for transcription factors SOX2 and NKX2-1 in sections from the proximal epithelium (broncho-esophageal fistula vs. trachea, respectively), bronchi, and distal bronchioles and alveoli. Scale bars represent 50 μm.

**Figure 3:**
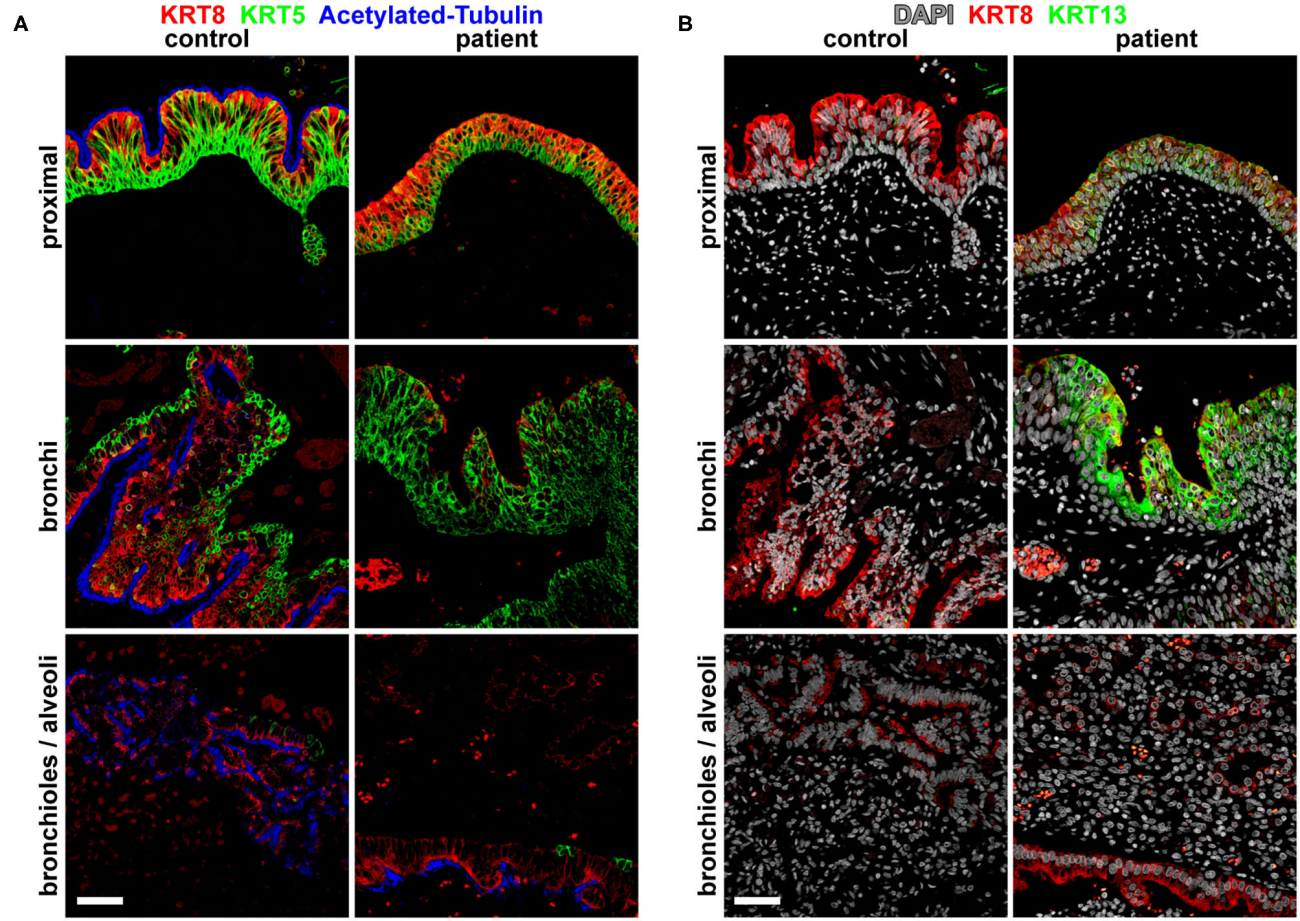
Molecular analysis for differentiation markers of the broncho-esophageal fistula and more distal airways. **(A)** Immunofluorescence staining for cytokeratins KRT5 and KRT8, as well as acetylated tubulin in sections from the proximal epithelium (broncho-esophageal fistula vs. trachea, respectively), bronchi, and distal bronchioles and alveoli. **(B)** Immunofluorescence staining for cytokeratins KRT8 and KRT13 in sections from the proximal epithelium (broncho-esophageal fistula vs. trachea, respectively), bronchi, and distal bronchioles and alveoli. Scale bars represent 50 μm.

Imaging analysis by cross-section and 3D reconstruction of the opacified lung suggested that although the airway track was malformed and connected to the distal esophagus, the pulmonary vasculature was grossly normal. Histological analysis of the surgically resected esophageal bronchus demonstrated an increased stratification of the epithelium proximally (fistula and bronchi) when compared to control proximal airway epithelium samples which were obtained postmortem from infant/neonatal patients without esophageal, tracheal, or lung abnormalities (Figure 2A). The distal airway epithelium (bronchioles and alveoli) of both control and patient tissue appeared to be similar (Figure 2A). Immunofluorescence staining for the transcription factors SOX2 (normally expressed in both the esophageal and proximal respiratory epithelia) and NKX2-1 (normally expressed only in the respiratory epithelium) suggested that the identity of the fistula and airways distal to the fistula was primarily respiratory (Figure 2B). However, additional staining for the cytokeratin proteins revealed heterogeneity in the fistula and more proximal airways of the current infant, which was not seen in the control tissue (Figure 3). In normal patients, the proximal airway epithelium is characterized by expression of keratin 5 (KRT5) and keratin (KRT8), which were abundant in the fistula/bronchial epithelium of this patient. However, the proximal airway of the current infant lacked acetylated tubulin, typically a marker of cilia in the airway epithelium, while the distal airways had similar expression patterns between patient and control tissue samples (Figure 3A). Additional staining for keratin (KRT13), a cytokeratin expressed in esophageal but not respiratory epithelium, revealed foci of the epithelium with some esophageal identity (Figure 3B). This suggested that the pathological tissue has an intermediate and heterogenous identity with lack of terminal differentiation.

Whole exome sequencing of the infant and his mother revealed rare, potentially deleterious single nucleotide variants with an allele frequency of <0.1% in gnomAD. Categorical assessment of these variants show they are in genes encoding cytoskeletal proteins, endosomal proteins, ubiquitination pathway proteins, and a gene related to Fanconi anemia complementation group (Supplementary Table 1). However, many variants were in genes that have not yet been described in tracheoesophageal or airway malformations. None of the variants were known to be pathogenic for the patient's congenital anomalies, and these will require further functional studies to causally link them to tracheal or esophageal defects.

## Discussion

Prior efforts to classify this relatively rare congenital defect have relied primarily on the anatomical configuration of the esophagus, fistula, and associated lung or lung lobe/segments (2, 6). Based on the work by Srikanth et al. in 1992, CBPFMs can be categorized into four main groups: group I occurs in association with an esophageal atresia/tracheoesophageal fistula (EA/TEF) where the distal esophagus has the additional fistula leading to a lung or lung lobe/segment; group II is the “esophageal lung,” where the aberrant connection is to the mainstem bronchus and thus sequesters the entire lung; group III describes the cases where the fistula connects the esophagus to a lobar or segmental bronchus; and group IV occurs when there is an abnormal fistula of either variety, but the normal connection with the main bronchus or trachea still exists (6).

We utilized UTE MRI to assess the esophageal and tracheal structures in the current patient. MRI is ideal for this application due to its clear imaging results without requiring ionizing radiation. Furthermore, UTE MRI is robust to motion due to its retrospective image reconstruction that allows for discarding of image data acquired during periods of patient motion, which obviates the need for sedation/anesthesia in a free-breathing infant.

This approach has significant potential benefits. First, the pre-operative anatomic information provided by 3D MRI renderings allow for surgical planning. Second, this approach allows for improved parental counseling regarding the type of defect and expected post-operative recovery. Finally, the ability to image patients both before and after repair provides the potential to evaluate the healing of the defect and the progression of other associated defects such as tracheomalacia and esophageal stricture. Based on our MRI evaluation, our patient has a group III CBPFM, which is the most common type of CBPFM reported.

In addition to anatomical variations of CBPFM, there are variations in the identity of the fistula/bronchus by histological analysis. Some cases report that the fistula/bronchial epithelium is of either esophageal (stratified squamous) or respiratory (columnar) epithelium, while other cases report that the identity is mixed but predominant in one type of epithelium (1–3, 7–9). For example, in one case described by Colleran et al. in 2017, they report that the epithelial lining of the bronchus was mixed, with predominantly stratified squamous epithelium and foci of ciliated columnar epithelium (2). However, our histological analysis suggests that our patient's fistula/bronchial epithelium appears to be pseudostratified columnar epithelium in the more distal regions and atypical stratified squamous epithelium more proximally.

In contrast to previous studies which only evaluated the basic tissue structure, our study adds molecular analysis of the esophageal bronchus and distal lung epithelium, which uncovers a more complex picture. Immunofluorescence staining of the proximal and distal epithelia for NKX2-1, a transcription factor expressed in respiratory epithelia, suggests that the entire epithelium is respiratory in nature. Further molecular analysis reveals that, both proximally and distally, there are patches of epithelia that express KRT13, a cytokeratin that is typically restricted in expression to stratified squamous epithelia. Our data suggest that this mixed phenotype was the result of early mispatterning of the esophageal vs. respiratory cell fates in the early foregut and improper segregation of the common fetal foregut tubes resulting in this aberrant connection. This is in contrast with the mechanical model of a re-connection and epithelial mixing of the early bronchus with the esophagus proposed by Srikanth et al. in 1992, as that model would not explain why all of the fistula/bronchial epithelium expresses the respiratory transcription factor NKX2-1 while focal patches also express the stratified squamous epithelial marker KRT13 (6).

Unfortunately, our whole-exome sequencing analysis could not pinpoint a single variant as the probable monogenic cause of this malformation. The primary limitation of our analysis was our inability to obtain and analyze the paternal sample to exclude the father's presumably non-deleterious variants. Our analysis assumes that the malformation is caused by a monogenic cause within the coding sequence of genes. Alternatively, it is also possible that this malformation is caused by mutations in the regulatory elements, multiple contributing loci, and/or environmental factors that contribute to this malformation.

Nevertheless, it is important to stress the associations of this rare foregut malformation with other congenital anomalies, particularly the VACTERL association. Although the patient in this case does not meet the typical criteria for this association, he had a horseshoe kidney. It has been noted that patients with CBPFMs may have other VACTERL anomalies or anomalies in DiGeorge's syndrome, particularly with EA/TEF as in group I CBPFMs (6, 10–12). Additionally, our patient's incidentally found malrotation reinforces previously described instances of malrotation that occur in some patients with CBPFM (9, 13). Therefore, in patients with any of these abnormalities, EA/TEF or CBPFM, a more thorough evaluation for other congenital defects may be warranted.

## Methods

### Human Tissue Samples

Deidentified control human tissue samples (from two different patients) were obtained from the Cincinnati Children's Hospital Medical Center Discover Together Biobank pathology department. These control samples were selected from postmortem samples of similar age (neonates/infants) with the absence of known lung/esophageal disease.

### Immunofluorescence Analysis

Paraffin slides were deparaffinized and subjected to antigen retrieval in 10 mM sodium citrate for 30 min prior to staining. Slides were then washed in phosphate-buffered saline (PBS) from Sigma-Aldrich (St. Louis, Missouri) and subsequently permeabilized with 0.5% TritonX-100 was from Promega (Madison, WI) in PBS for 10 min, blocked in 5% normal donkey serum (Jackson ImmunoResearch, West Grove, PA, USA) for 1 h, and incubated in primary antibody overnight at 4°C. On the next day, slides were thoroughly washed in 0.3% TritonX-100 in PBS, incubated in secondary antibody (at 1:500) for 1 h, and then thoroughly washed again. Primary antibodies used included goat anti-SOX2 (1:100, Santa Cruz Biotechnology #sc-17320, Dallas, TX, USA), rabbit anti-NKX2-1 (1:500, Abcam #ab-76013, Cambridge, UK), rat anti-Krt8 (1:100, DSHB TROMA-I, Iowa City, IA, USA), rabbit anti-KRT13 (1:500, Abcam ab92551, Cambridge, UK), chicken anti-KRT5 (1:500, Biolegend #905901, San Diego, CA, USA), mouse anti-acetylated tubulin (1:500, Sigma T6793, St. Louis, MO, USA). Secondary antibodies were used at a 1:500 concentration; these include AlexaFluor donkey anti-mouse 647 (ThermoFisher #A31571, Waltham, MA, USA), AlexaFluor donkey anti-rabbit (ThermoFisher #A10040, Waltham, MA, USA), AlexaFluor donkey anti-goat 568 (ThermoFisher #A10037, Waltham, MA, USA), AffiniPure donkey anti-rat 647 (Jackson Immunoresearch Labs #712-605-153, West Grove, PA, USA), and AffiniPure donkey anti-chicken (Jackson Immunoresearch Labs, #703-545-155, West Grove, PA, USA).

### Exome Sequencing and Analysis

Exome sequencing and analysis on the proband and mother were performed as previously described (14).

## Data Availability Statement

The original contributions presented in the study are included in the article/Supplementary Material, further inquiries can be directed to the corresponding author/s.

## Ethics Statement

The studies involving human participants were reviewed and approved by Cincinnati Children's Hospital Medical Center Institutional Review Board. Written informed consent to participate in this study was provided by the participants' legal guardian/next of kin. Written informed consent was obtained from the minor(s)' legal guardian/next of kin for the publication of any potentially identifiable images or data included in this article.

## Author Contributions

ST, DK, TN, AZ, and JMW participated in the immunofluorescence analysis. NH and JCW aided with the UTE MRI image analysis and 3D reconstruction. WC performed the whole exome sequencing analysis. PK supervised the data collection. ST and PK drafted, reviewed, and revised the manuscript. All authors critically reviewed the manuscript and approve the final manuscript.

## Conflict of Interest

The authors declare that the research was conducted in the absence of any commercial or financial relationships that could be construed as a potential conflict of interest.

## Supplementary Material

Supplementary Table 1 Candidate deleterious variants in candidate genes for patient with broncho-esophageal fistula. Type of mutations: “Stop” indicates stop codon introduced, “mis” indicates missense variant, “splice” indicates splice variant, and “frame” indicates frameshift variant.
