# Supplementary material for: Case Report: Esophageal Bronchus in a Neonate, With Image, Histological, and Molecular Analysis
Source: Front Pediatr. 2021 Jul 9;9:707822. doi: 10.3389/fped.2021.707822 (PMC8298819; doi:10.3389/fped.2021.707822)
Supplement: Supplementary Table 1 — Candidate deleterious variants in candidate genes for patient with broncho-esophageal fistula. Type of mutations: “Stop” indicates stop codon introduced, “mis” indicates missense variant, “splice” indicates splice variant, and “frame” indicates frameshift variant. [file Table_1.DOCX]

| **Gene** | **Chr** | **Position** | **ref > alt** | **Variant** | **Type** | **pLI** | **REVEL** | **CADD** | **Associated OMIM condition [inheritance pattern]** |
| --- | --- | --- | --- | --- | --- | --- | --- | --- | --- |
| EPB41L4A | 5 | 112209938 | C > A | ENSP00000482810.1:p.Glu378Ter | stop | 1.99E-11 | 0 | 37 |  |
| FAM200A | 7 | 99547799 | C > T | ENSP00000411372.1:p.Trp203Ter | stop | 6.01E-05 | 0 | 35 |  |
| TSC22D1 | 13 | 44575815 | A > C | ENSP00000397435.2:p.Leu87Arg | mis | 0.9412 | 0.304 | 33 |  |
| DOCK6 | 19 | 11233292 | G > A | ENSP00000294618.6:p.Arg877Cys | mis | 2.83E-19 | 0.358 | 32 | Adams-Oliver syndrome 2 [AR] |
| KIAA1328 | 18 | 36959323 | A > G | ENSP00000280020.5:p.Tyr155Cys | mis | 6.07E-05 | 0.421 | 29.5 |  |
| TMPRSS12 | 12 | 50843023 | C > G | ENSP00000381476.3:p.Ser20Ter | stop | 0.003116 | 0 | 28.6 |  |
| CSMD1 | 8 | 3493624 | C > T | ENSP00000489225.1:p.Val483Met | mis & splice | 0 | 0.197 | 28.3 |  |
| EPB41L4B | 9 | 109208046 | C > T | ENSP00000363694.3:p.Glu586Lys | mis | 0.9997 | 0.695 | 26.4 |  |
| PHACTR2 | 6 | 143678208 | C > G | ENSP00000417038.2:p.Ser15Arg | mis & splice | 0.007389 | 0.107 | 26.3 |  |
| ACTN1 | 14 | 68874953 | G > A | ENSP00000377941.4:p.Ala884Val | mis | 0.9999 | 0.245 | 25.6 | Bleeding disorder, platelet-type 15 [AD] |
| TATDN2 | 3 | 10249298 | C > T | ENSP00000287652.4:p.Ala33Val | mis | 0.000743 | 0.122 | 25.6 |  |
| MPZL3 | 11 | 118233459 | C > G |  | splice | 6.52E-09 | 0 | 25.1 |  |
| MAPK8IP3 | 16 | 1765113 | C > T | ENSP00000481780.1:p.Pro794Leu | mis | 1 | 0.372 | 24.8 | Neurodevelopmental disorder with variable brain abnormalities [AD] |
| DIP2B | 12 | 50686606 | C > A | ENSP00000301180.5:p.Ser492Tyr | mis | 0.9997 | 0.083 | 24.7 | Mental Retardation, FRA12A type [AD] |
| LUC7L | 16 | 189977 | G > A | ENSP00000293872.8:p.Ala322Val | mis | 0.1642 | 0.045 | 24.7 |  |
| TBC1D30 | 12 | 64866880 | A > C | ENSP00000440207.1:p.Gln423Pro | mis | 0 | 0.315 | 24.2 |  |
| AC068946.2 | 2 | 219224659 | C > A |  | mis | 0.7148 | 0.652 | 23.6 |  |
| SRFBP1 | 5 | 122020091 | C > T | ENSP00000341324.4:p.Ala119Val | mis | 8.19E-05 | 0.488 | 23.2 |  |
| SAT2 | 17 | 7630753 | G > T |  | mis | 0.000189 | 0.357 | 23.1 |  |
| MGA | 15 | 41762207 | A > G | ENSP00000219905.7:p.Lys2530Arg | mis | 1 | 0.269 | 23.1 |  |
| NYAP1 | 7 | 100490535 | G > A | ENSP00000300179.2:p.Arg655Gln | mis | 0.9909 | 0.031 | 23.1 |  |
| ZCCHC3 | 20 | 297824 | G > A | ENSP00000484056.1:p.Gly80Ser | mis | 0 | 0.024 | 23 |  |
| CAD | 2 | 27222302 | A > G | ENSP00000264705.3:p.Asn154Ser | mis | 1 | 0.576 | 22.9 | Epileptic Encephalopathy, early infantile [AR] |
| KMT2C | 7 | 152181360 | C > T | ENSP00000262189.6:p.Arg2167Gln | mis | 1 | 0.49 | 22.9 | Kleefstra syndrome 2 [AD] |
| MFSD2A | 1 | 39968604 | C > T | ENSP00000361895.5:p.Pro476Leu | mis | 0.9177 | 0.679 | 22.8 | Microcephaly 15 [AR] |
| NUP155 | 5 | 37331687 | G > C | ENSP00000231498.3:p.Gln543Glu | mis & splice | 0.9906 | 0.286 | 22.8 | Atrial fibrillation [AR] |
| AP3D1 | 19 | 2120980 | C > T | ENSP00000495274.1:p.Ala455Thr | mis | 0.798 | 0.29 | 22.7 | Hermansky-Pudlak Syndrome 10 [AR] |
| RPL10 | X | 154400837 | C > T | ENSP00000413436.2:p.Arg210Trp | mis | 0.9012 | 0.122 | 22.7 | Autism and Mental Retardation [X-linked] |
| LUM | 12 | 91108157 | T > C | ENSP00000266718.4:p.Asn275Asp | mis | 0.1019 | 0.063 | 22.7 |  |
| EPPK1 | 8 | 143869286 | G > A | ENSP00000484472.1:p.Ala1323Val | mis | 4.95E-21 | 0.256 | 22.6 |  |
| MROH1 | 8 | 144239666 | G > A | ENSP00000435565.1:p.Arg562His | mis | 0.5871 | 0.135 | 22.5 |  |
| CDK12 | 17 | 39471584 | G > T | ENSP00000398880.3:p.Leu584Phe | mis | 1 | 0.06 | 22.3 |  |
| DAPL1 | 2 | 158807089 | G > A | ENSP00000309538.3:p.Asp61Asn | mis | 0.000268 | 0.04 | 22.3 |  |
| NUP155 | 5 | 37303290 | C > T | ENSP00000231498.3:p.Arg1096His | mis | 0.9906 | 0.322 | 22.1 |  |
| KIRREL3 | 11 | 126526604 | C > T | ENSP00000435466.2:p.Glu73Lys | mis | 0.9775 | 0.204 | 22 | variant of unknown significant (prev. associated with Mental Retardation) |
| TRPV6 | 7 | 142876787 | G > A | ENSP00000352358.4:p.Arg220Trp | mis | 0.001414 | 0.304 | 21.9 | transient neonatal hyperparathyroidism [AD] |
| VAX1 | 10 | 117134412 | C > T | ENSP00000358207.4:p.Ala201Thr | mis | 0.535 | 0.212 | 21.3 | microphthalmia [AD] |
| TSR3 | 16 | 1349585 | C > A | ENSP00000007390.2:p.Ser264Ile | mis | 0.002408 | 0.154 | 21.1 |  |
| OR52K1 | 11 | 4489607 | G > A | ENSP00000493011.1:p.Arg236His | mis | 1.76E-05 | 0.058 | 21.1 |  |
| SPEM1 | 17 | 7421015 | C > T | ENSP00000315554.3:p.Arg114Cys | mis | 0.0235 | 0.141 | 20.8 |  |
| HCN3 | 1 | 155291760 | T > A |  | mis | 1E-05 | 0.545 | 20.6 |  |
| DNASE1L3 | 3 | 58192794 | C > T | ENSP00000378053.2:p.Val271Ile | mis | 0.1555 | 0.131 | 20.2 | SLE [AR] |
| QSOX1 | 1 | 180194273 | G > A | ENSP00000356574.3:p.Arg450Gln | mis | 6.78E-05 | 0.099 | 20.2 |  |
| KRTAP4-3 | 17 | 41167832 | C > T | ENSP00000375151.2:p.Cys114Tyr | mis | 0.000537 | 0.064 | 19.13 |  |
| DYSF | 2 | 71660584 | A > G | ENSP00000386881.3:p.Ile1646Val | mis | 1E-15 | 0.078 | 18.9 | muscular dystrophy, myopathy [AR] |
| SLC12A9 | 7 | 100857107 | T > C | ENSP00000275730.4:p.Ser230Pro | mis | 2.37E-06 | 0.516 | 18.57 |  |
| CNTNAP1 | 17 | 42686136 | A > T | ENSP00000264638.3:p.Thr299Ser | mis | 0.004109 | 0.269 | 18.55 | lethal congenital contracture [AR] |
| SLX4 | 16 | 3606492 | C > T | ENSP00000294008.3:p.Glu248Lys | mis | 8.9E-08 | 0.064 | 18.38 | Fanconi Anemia [AR] |
| VWA8 | 13 | 41833395 | G > A | ENSP00000368612.3:p.Ala521Val | mis | 0.000293 | 0.069 | 18.18 |  |
| KIF13A | 6 | 17796727 | C > T | ENSP00000259711.6:p.Gly962Ser | mis | 1 | 0.246 | 18.1 |  |
| CDH15 | 16 | 89192237 | G > A | ENSP00000289746.2:p.Val550Ile | mis | 5.96E-12 | 0.038 | 17.95 | Mental Retardation [AD] |
| PTPRQ | 12 | 80459359 | G > A | ENSP00000482885.1:p.Arg179Gln | mis | 0 | 0 | 16.26 | deafness [AR] |
| ARHGEF7 | 13 | 111217884 | A > G |  | mis | 0.954 | 0.033 | 15.78 |  |
| ZNF442 | 19 | 12350640 | G > GT | ENSP00000242804.4: p.His315GlnfsTer8 | frame | 1.47E-07 | 0 | 0 |  |
| MAGIX | X | 49165199 | C > G | ENSP00000471299.1:p.Leu173Val | mis | 0.003592 | 0 | 17.5 |  |
| SYNE2 | 14 | 64031298 | G > A | ENSP00000350719.3:p.Glu2388Lys | mis | 1.85E-11 | 0.071 | 17.26 | Emery-Dreifuss Muscular Dystrophy [AD] |
| TULP4 | 6 | 158489711 | G > A | ENSP00000356064.3:p.Ser537Asn | mis | 0.9999 | 0.073 | 22.5 |  |
| TULP4 | 6 | 158503555 | G > A | ENSP00000356064.3:p.Asp1298Asn | mis | 0.9999 | 0.225 | 21.6 |  |
| BOP1 | 8 | 144263733 | C > T | ENSP00000455106.1:p.Arg417Gln | mis | 0.5268 | 0.166 | 23.7 |  |
| MROH1 | 8 | 144239666 | G > A | ENSP00000435565.1:p.Arg562His | mis | 0.5871 | 0.135 | 22.5 |  |
| OTOG | 11 | 17638533 | C > G | ENSP00000382323.2:p.Cys2638Trp | mis | 0 | 0.552 | 29.8 | Deafness [AR] |
| OTOG | 11 | 17635125 | G > A | ENSP00000382323.2:p.Arg2556Gln | mis | 0 | 0.061 | 23.8 |  |
